# The Alterations of Vaginal Microbiome in HPV16 Infection as Identified by Shotgun Metagenomic Sequencing

**DOI:** 10.3389/fcimb.2020.00286

**Published:** 2020-06-23

**Authors:** Qian Yang, Yaping Wang, Xinyi Wei, Jiawei Zhu, Xinyu Wang, Xing Xie, Weiguo Lu

**Affiliations:** ^1^Women's Reproductive Health Laboratory of Zhejiang Province, Women's Hospital, Zhejiang University School of Medicine, Hangzhou, China; ^2^Department of Gynecologic Oncology, Women's Hospital, Zhejiang University School of Medicine, Hangzhou, China

**Keywords:** vaginal microbiome, HPV infection, cervical cancer, shotgun metagenomic sequencing, metabolism

## Abstract

The association of microbiome imbalance with cancer development is being one of the research hotspots. Persistent HPV infection is a causal event in cervical cancer initiation, but, little is known about the microbiome composition and function in HPV infection. Here we identified the compositional and functional alterations on vaginal samples from 27 HPV16 positive women and 25 age-matched HPV negative controls using shotgun metagenomic sequencing, to provide a comprehensive investigation describing the microbial abundances and enriched metabolic functions in cervicovaginal metagenomes. We further employed qPCR assays to evaluate two selected gene markers of HPV16 infection in an independent validation cohort consisting of 88 HPV16 positive women and 81 controls, and six selected species markers in a subset of validation cohort of 45 HPV16 positive women and 53 controls. We found that the relative abundance of dominant Firmicutes was lower, Actinobacteria, Fusobacteria and viruses phyla were significantly higher in the HPV16-positive group; 77 genera including *Gardnerella, Peptostreptococcus*, and *Prevotella* were higher, and 20 genera including *Lactobacillus* and *Aerococcus* were lower in the HPV16-positive women. Abundance of 12 genes, 17 genera, and 7 species biomarkers showed an excellent predictive power for the HPV16-positive individuals, with 0.861, 0.819, and 0.918, respectively, of the area under the receiver-operating characteristic curve (AUC). We further characterized the microbial function, and revealed that HPV16-positive women were enriched in metabolism and membrane transport, and depleted by glycan biosynthesis and metabolism, and replication and repair. Quantitative PCR measurements validated that one gene marker and three species were significantly enriched in HPV16-positive women. These results highlight a fundamental fact that there are altered composition and function of the vaginal microbiome in HPV16-positive women, suggesting that vaginal dysbiosis may be associated with HPV infection in the female genital tract.

## Introduction

Persistent high-risk human papillomavirus (HPV) infection is the central causal agent of cervical intraepithelial neoplasia (CIN) and cervical carcinoma (Walboomers et al., 1999). HPV16 is the most common type in cases (Bruni et al., 2010; Sanjose et al., 2010). However, only a few women with HPV infection progress to cervical cancer, and more than 90% of viruses are cleared within 6–18 months. Although lifetime risk of acquiring any type of HPV infection exceeds 80%, HPV persistent infection occurs in nearly 10% of infected women (Stanley, 2010). The reasons for high-risk HPV persistent infection in some women but not others are still unknown. Some researches point out that individual differences in immune function (Shannon et al., 2017) or genetic susceptibility (Zou et al., 2016) may play a role, but the determining factor may also lie in the cervicovaginal microenvironment where the cervix is located.

The microenvironment in the female reproductive tract is composed of anatomical structure, endocrine system, local immunity, and vaginal microbiota. Rapidly emerging evidences reveal that healthy vaginal microbiota is an essential component of a multifaceted defense system that operates to protect women against disease (Gajer et al., 2012; Gopinath and Iwasaki, 2015; Anahtar et al., 2018; Kroon et al., 2018). As an indispensable defense system in cervicovaginal microenvironment, the vaginal microbiome has recently drawn considerable attention for its potential role in the female genital diseases, such as reproductive tract infection (RTI), spontaneous preterm delivery (Freitas et al., 2018; Elovitz et al., 2019), and preterm fetal membrane rupture (Brown et al., 2018), although a causal relationship has yet to be established.

High-risk HPV, as a foreign pathogen, may interact with the cervicovaginal microbiome inevitably once infection occurs, causing microbiome dysbiosis. Or vice versa, maybe the dysbiosis predisposes individuals to HPV infection. Nevertheless, the microbiome dysbiosis could be more suitable for HPV infection, even persistent infection. Previous studies using 16S rRNA gene sequencing have revealed that individuals with HPV infection have higher microbial diversity with a lower proportion of *Lactobacillus spp*. (Lee et al., 2013; Shannon et al., 2017), and *Sneathia spp*. may act as a possible microbiological marker associated with HPV infection. However, 16S rRNA gene amplification ignores microbes that lack a gene to match the universal primers, like eukaryotes, viruses, and archaea that are not accounted for (Ranjan et al., 2016). Thus, explanation of results generated by 16S rRNA gene sequencing may be confined by its low taxonomical and functional resolution. The use of shotgun metagenomic sequencing allows to identify bacterial taxa to species level (Shah et al., 2018) and to analyze vaginal microbiota functions without reliance on prediction. Hence, whole genomic DNA-based sequencing is more suitable than 16S rRNA gene-based methods for exploration of the association between the vaginal microbiome and HPV infection.

In the present study, we used shotgun metagenomics profiling of HPV16-positive and HPV-negative vaginal microbiota to screen potential microbiological biomarkers in cohorts, and independently validated them using an affordable technology that can translate to clinical practice. The study aims to explore the association between vaginal dysbiosis and HPV infection in the female genital tract.

## Materials and Methods

### Study Population and Sampling

All samples derived from 2251 non-pregnant, reproductive-age women who went to colposcopy clinic after cervical cancer screening using HPV testing and cytology during 2017.2–2018.11. All women aged 25–50, had regular menses and sexual activities without using hormonal contraception. Those women were excluded if they met any of the following criteria: (1) use of antibiotics or vaginal antimicrobials (oral pills or by topical application in vulvar/vaginal area) in the past month, and vaginal intercourse or vaginal lavage within the last 3 days; (2) history of BV, candidiasis, urinary tract infections, or active sexual transmitted diseases (STD, specifically chlamydia, gonorrhea, syphilis, genital herpes, trichomoniasis) within the previous 2 months; (3) history of hysterectomy, cervical cold knife conization (CKC) or loop electrosurgical excision procedure (LEEP); (4) history of systemic diseases such as diabetes, autoimmune disease, and malignant tumors.

**Figure 1:**
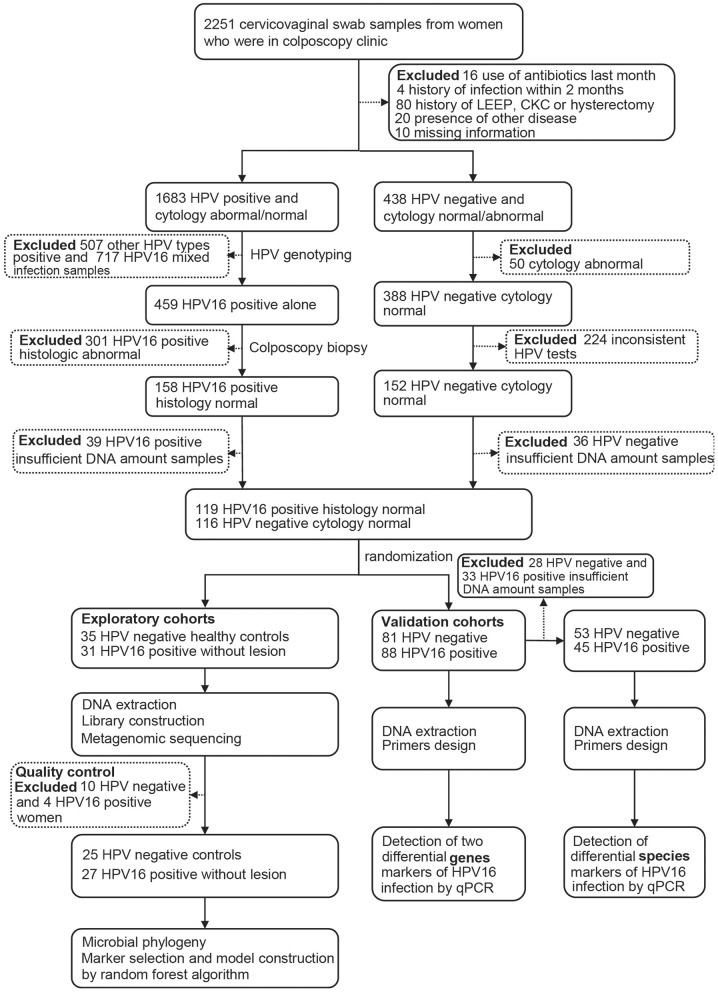
Flowchart of the study.

After completing a clinical and sexual behavior questionnaire, vaginal secretion of women was obtained before cervical exfoliated cell sampling for further investigation. Four sterile swab samples were taken from near the vaginal fornix and cervix, placed into a sterile tube, stored in liquid nitrogen immediately, and archived at −80°C until further analysis. Women with abnormal cytology and/or positive HPV testing underwent colposcopy with or without biopsy, and those HPV-positive samples were genotyped, and only those with HPV16 positive and confirmed histologically to be normal were, as HPV16-positive group, included in the study. Women with HPV negative and normal cytology were included as controls. The study flowchart is illustrated in Figure 1.

All participants provided written informed consent and the study was approved by the Ethics Committee of the Hospital.

### HPV Test and Genotyping

All HPV positive samples were genotyped using HybriBio's proprietary flow-through hybridization technique with Hybribio Rapid GenoArray test kit (GA), which can identify 6 low-risk types (6, 11, 42, 43, 44, and CP8304) and 15 high-risk types (16, 18, 31, 33, 35, 39, 45, 51, 52, 56, 58, 59, 68, 66, and 53). All the tests were performed according to the manufacturer's protocols. Only HPV16 single positive samples were selected for the study, including 31 in exploratory cohorts and 88 in validation cohorts. Totally 116 individuals with two consistent HPV negative results were selected as controls, including 35 in exploratory cohorts and 81 in validation cohorts.

### DNA Extraction and Metagenomic Sequencing

Qiagen QIAmp DNA Microbiome Kit (Qiagen) was used for DNA extraction according to the manufacturers' instructions. DNA concentration was measured by Qubit®2.0 (Invitrogen, U.S.) and its integrity and molecular size were estimated by 1% agarose gel electrophoresis containing 0.5 mg/ml ethidium bromide. NEBNext® Ultra™ DNA Library Prep Kit for Illumina® was used for library construction. The quality of the library was evaluated using Agilent 2100 (Agilent, U.S). All samples constructed the libraries were pooled and sequenced on the Hiseq X-ten platform (Illumina, San Diego, CA). The raw reads were cleaned by removing low-quality sequences (reads with unknown bases “N”), reads with more than 20% low-quality bases and <60% high-quality bases. Then, clean reads were aligned against all known microbial genomes, as downloaded from the National Center for Biotechnology Information GenBank with SOAPaligner (version 2.21) (Li et al., 2008) and the reads that mapped to the host genome were abandoned. The subsequent reads were selected for further analysis.

### Taxonomic and Gene Profiling

Clean reads were aligned with the NCBI database for the detection of known bacteria, fungi, viruses, and archaea. Then, the aligned reads were classified as Kingdom, Phylum, Class, Order, Family, Genus, Species to count classification and abundance. The taxonomy profile was constructed at different levels. Preprocessed reads were assembled by SOAPdenovo (Version 1.05) (Luo et al., 2012) to acquire the high-quality reads from each sample into contigs. Software MetaGeneMark (Noguchi et al., 2006) was used to predict genes in the assembled scaffolds with default parameters. The predicted open reading frames (ORFs) were compared against the NCBI non-redundant sequence database using BLAST. To obtain a non-redundant gene catalog, pairwise comparison of predicted ORFs were carried out with coverage ≥90% and identity ≥95%. Clean paired reads were aligned with the genes in the non-redundant catalog. The calculation formula of gene abundance used was from the study by Qin et al. (2014). Functional annotations were performed by blast against the Kyoto Encyclopedia of Genes and Genomes (KEGG) (Kanehisa et al., 2004). The assembled genes were also clustered according to function on different levels.

### Data Analysis

The non-parametric Wilcox rank-sum test (wilcox.test in R) was employed to analyze the statistical significance of the different taxonomic (phylum, genus, species) levels, gene and KO (KEGG orthologs) between HPV16-positive women and healthy controls. The Benjamini-Hochberg method (false discovery rate, FDR) was used for correction in multiple testing in which a *P* < 0.05 was considered significant. Enriched features with an adjusted *P* < 0.05 were identified, and the enrichment group was then determined according to a higher mean rank-sum value. To determine the features (organisms, KOs) most likely to explain the differences between the HPV16-positive women and healthy controls, we applied Linear discriminant analysis (LDA) effect size (LEfSe) analysis (Segata et al., 2011) with an LDA score cut-off of 2.0 and the Metastats software (White et al., 2009), respectively.

### Validation of Microbial Markers by qPCR

TaqMan quantitative PCR was chosen to estimate the abundances of selected gene and species markers in vaginal samples. Primer sequences were designed manually and identified using agarose gel electrophoresis after PCR amplification for determination of product size and possible secondary structures (Supplementary Table 5). Quantitative PCR was performed on an ABI2720 Real-Time PCR System using SYBR Green qPCR Master Mix (TaKaRa DRR041A). Universal 16S rRNA gene was used as internal control and abundance of gene and species markers were expressed as relative levels to 16S rRNA gene.

## Results

### Cohort Demographics

Totally 52 women, including 27 HPV16 positive women and 25 HPV negative controls, were enrolled in the study. The demographic characteristics of both groups were summarized in Table 1. There were no differences in demographics between the two groups, including age, BMI, menarche age and days since last menstrual period, nor were there differences in contraceptive methods, age at first sexual intercourse, sexual frequency, number of sexual partners and contraceptive methods. In order to validate the findings of sequencing, a validation cohort was recruited, including 88 HPV16 positive and 81 HPV negative women (Figure 1 and Supplementary Table 1). Also, there were no significant differences in demographics between the two groups in the validation cohort, except for the number of sexual partners (p=0.007, Pearson Chi-Square test).

**Table 1:** Socio-demographic of subjects in exploratory cohort.

**Characteristics**	**NC group (*n* = 25)**	**HPV16+ group (*n* = 27)**	***P*-value^a^**
Age (mean ± SD, range)	36.9 ± 4.9 (27–42)	34.5 ± 4.5 (25–41)	0.075
BMI (mean ± SD, range)^b^	21.71 ± 2.5 (17.8–28.0)	21.3 ± 2.7 (16.8–28.0)	0.561
Smoker(s)	0	2/27	0.491
Passive smokers	10/25	11/27	0.956
Menarche age	14.5 ± 1.5 (11–18)	14.4 ± 1.2 (12–16)	0.789
Time since last menstrual period (mean ± SD)	15.7 ± 5.7	16.7 ± 7.1	0.568
Contraceptive method for nearly 1 year			0.638
Condom	13	15	
IUD^c^	4	2	
Tubal ligation	4	3	
No contraception	4	7	
Hepatitis B surface antigen-positive	2	2	
Age of first sexual life	21.6 ± 2.0 (18–25)	21.2 ± 2.6 (17–29)	0.483
Sexual frequency (weekly)			0.440
≤ 1 time/week	14	17	
2–3 times/week	8	6	
≥4 times/week	0	2	
Unknown	3	2	
Lifetime number of sexual partners			0.272
1	17	12	
2	4	5	
3–4	3	9	
Unknown	1	1	

a *Student's t-test, Pearson Chi-Square test and Fisher's exact test adapted to the variable distribution*.

b *BMI, body mass index*.

c *IUD, intrauterine device*.

### Phylogenetic and Gene Profiles of Vaginal Microbiota in HPV-16 Positive Women and Controls

In 52 exploratory subjects, 389 million 150 bp paired-end high-quality reads free of adaptor, low quality, and human DNA contaminants were obtained after quality control, with an average of 7.49 million clean reads per sample for microbial taxonomic classification (Supplementary Table 2). Rarefaction analysis showed curves reaching the plateau, suggesting that the sequencing depth covered most prevalent microbial genes in samples (Supplementary Figure 1A).

In terms of alpha diversity, the Shannon-wiener and the Simpson indexes did not show a significant difference (Supplementary Figure 1B). Comparing the taxonomy between HPV16-positive women and controls, we found that 905 genera were shared in two groups, but 169 were only in HPV16-positive women and 140 genera were only in controls. Similar results were observed at species level. Totally 3591 species were shared in both groups, whereas 773 species were only in HPV16-positive women and 653 only in controls (Supplementary Figure 1C). We performed a principal component analysis (PCA) based on gene profiles, but did not find a significant difference between HPV16-positive women and controls (*p* = 0.092, *r* = 0.035, Adonis test) (Supplementary Figure 1D).

### Taxonomic Alterations of Vaginal Microbiota in HPV16 Positive Women

**Figure 2:**
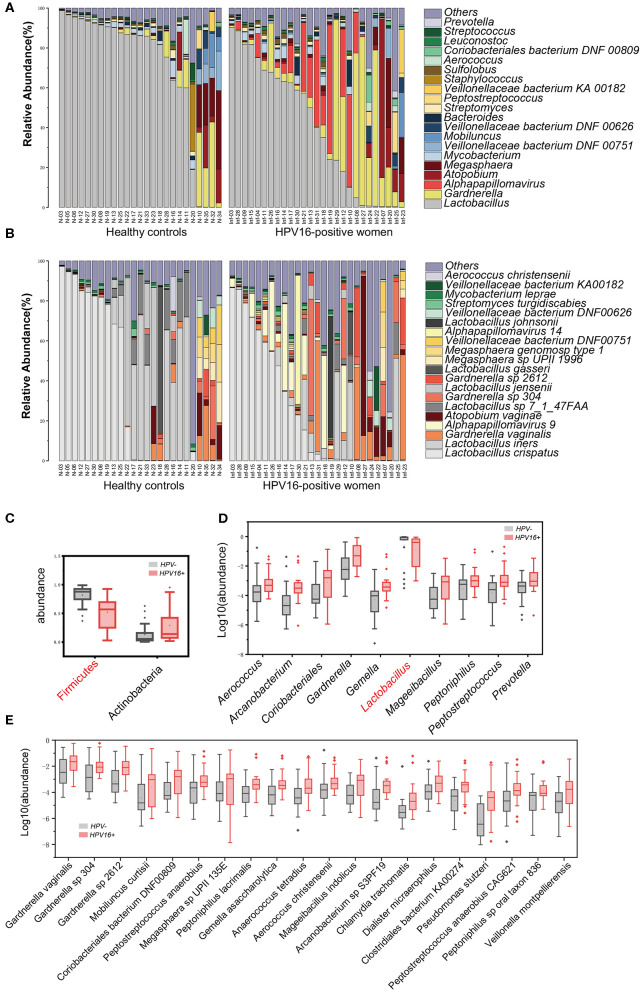
Phylogenetic profiles in vaginal microbes between HPV16-positive women and controls. Composition of vaginal microbiota in two groups at the genus level **(A)** and species level **(B)**. Comparison of differentially abundant phylotypes identified by the Wilcoxon rank-sum test, at phyla **(C)**, genera **(D)**, and species **(E)** level, respectively. Only the top 2 phyla, top 10 genera and top 20 species are shown. The phylotypes enriched in the control group are colored with red. The relative abundances are shown by boxplot. Boxes represent the interquartile ranges, lines inside the boxes denote medians, and “+” denotes means.

To illustrate the phylogenetic profiles in vaginal microbes, we aligned the clean reads with the NCBI database. We herein found that bacteria were the major organism identified in the vaginal microbes, with few fungal organisms at low relative abundance (Supplementary Table 2). Although the composition of individual species varies, we still observed similarities within groups. The genus *Lactobacillus*, followed by *Gardnerella*, was overwhelmingly dominated in the vaginal flora at the genus level, in both HPV16-positive and control groups. Besides, *Atopobium, Megasphaera, Mycobacterium*, and *Veillonellaceae* were found relatively more often (Figure 2A). Similarly, at species level, *Lactobacillus crispatus, Lactobacillus iners*, and *Gardnerella vaginalis* were the top three species in both groups, followed by *Alpha pillomavirus* 9, *Atopobium vaginae*, etc. *Alpha papillomavirus* 9 enriched in HPV16-positive group was expected, because HPV16 belongs to *Alpha papillomavirus* 9 (Figure 2B).

Further, all 52 subjects were divided into two types: lactobacilli accounted for at least 50% of the species (community type L) and lactobacilli accounted for <50% of the species present (community type O) (Nené et al., 2019). We found that 15/27 (55.6%) in HPV16-positive women and 20/25 (80%) in controls were type L, 12/27 (44.4%) in HPV16-positive and 5/25 (20%) in controls were type O (*p* = 0.060, Pearson Chi-square test, data not shown). The results suggest that a lower proportion of lactobacilli is more common in HPV16 positive women.

To identify microbial taxa contributing to the dysbiosis, we examined taxonomic differences between controls and HPV16-positive group, and found that the abundance of phyla Actinobacteria (*p* = 0.00803, Wilcoxon rank-sum test), Fusobacteria (*p* = 0.010), and viruses (*p* = 0.0006) were significantly higher, while Firmicutes was significantly lower in the HPV16-positive group (Figure 2C and Supplementary Table 3). Consistent with the phylum level analysis, genus *Gardnerella*, belongs to Actinobacteria, was also increased, while *Lactobacillus*, belonging to Firmicutes, was decreased in HPV16-positive women (Figure 2D). Also, genus, like *Peptostreptococcus* and *Prevotella*, were enriched in HPV16-positive group. However, such differences at phylum and genus levels did not appear in dominant species, such as *L. crispatus* and *L. iners*, while mainly appeared in non-dominant species, for instance, *Gardnerella vaginalis* (*p* = 0.0172), *Gardnerellasp_304* (*p* = 0.0022), and *Gardnerella sp_2612* (*p* = 0.0011) were enriched in HPV16-positive women. And some opportunistic pathogens like *Mobiluncus curtisii* (*p* = 0.0106), *Coriobacteriales bacterium DNF00809* (*p* = 0.0050), *Peptostreptococcus anaerobius* (*p* = 0.0067), *Veillonella montpellierensis* (*p* = 0.0019), and *Megasphaera sp UPII_135E* (*p* = 0.0352) were significantly enriched in HPV16-positive women (Figure 2E and Supplementary Table 3). Our results suggest that the differences between the two groups may depend on not only classical dominant species, but also the opportunistic pathogens or non-dominant species.

### HPV16 Infection Biomarker Discovery

**Figure 3:**
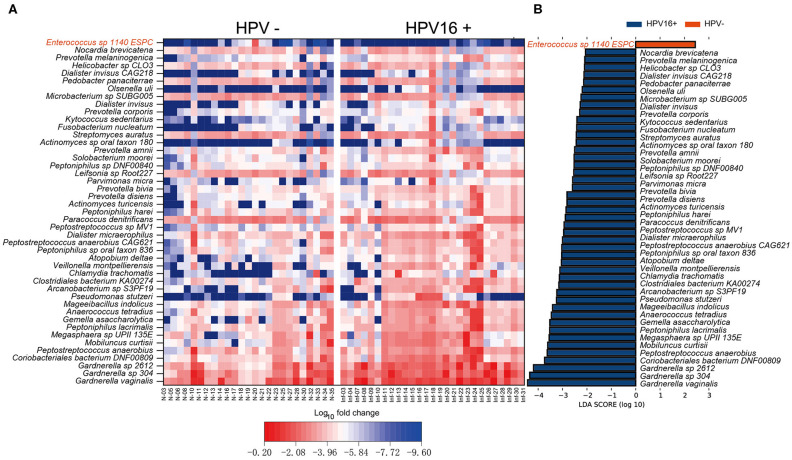
Vaginal microbiome as HPV16-infection markers. **(A)** Heatmap showing the relative fold change of bacterial species in HPV16 infection. The species enriched in controls are colored with red. **(B)** Histogram of the LDA scores computed for species differentially abundant between HPV16-positive women and controls. The LDA scores (log10) > 2 are listed.

To define potential metagenomic biomarkers that could be more likely to explain the differences between the HPV16-positive and control groups, we performed Linear Discriminant Analysis (LDA) Effect Size (LEfSe) analysis. To exclude the influences of HPV bringing to differences, here we only presented the results using bacteria taxonomy. Forty-four species biomarkers were enriched in HPV16-positive women while only one was enriched in controls (Figure 3). Meanwhile, analysis of fold change against mean abundance showed increased and decreased HPV16-specific LEfSe biomarkers intuitively (Figure 3A). In agreement with previous studies (Brotman et al., 2014; Di Paola et al., 2017; Chen et al., 2019), we found significant altered opportunistic pathogens in HPV16-positive women, including increased *Gardnerella vaginalis, Gardnerella_sp_304*, and *Gardnerella_sp_2612* (*Gardnerella* genus), *Peptostreptococcus anaerobius, Mobiluncus curtisii, Prevotella disiens, Prevotella bivia, Prevotella amnii, Prevotella corporis* (*Prevotella* genus)*, Fusobacterium nucleatum* and decreased *Enterococcus sp_1140_ESPC* (Figure 3B).

**Figure 4:**
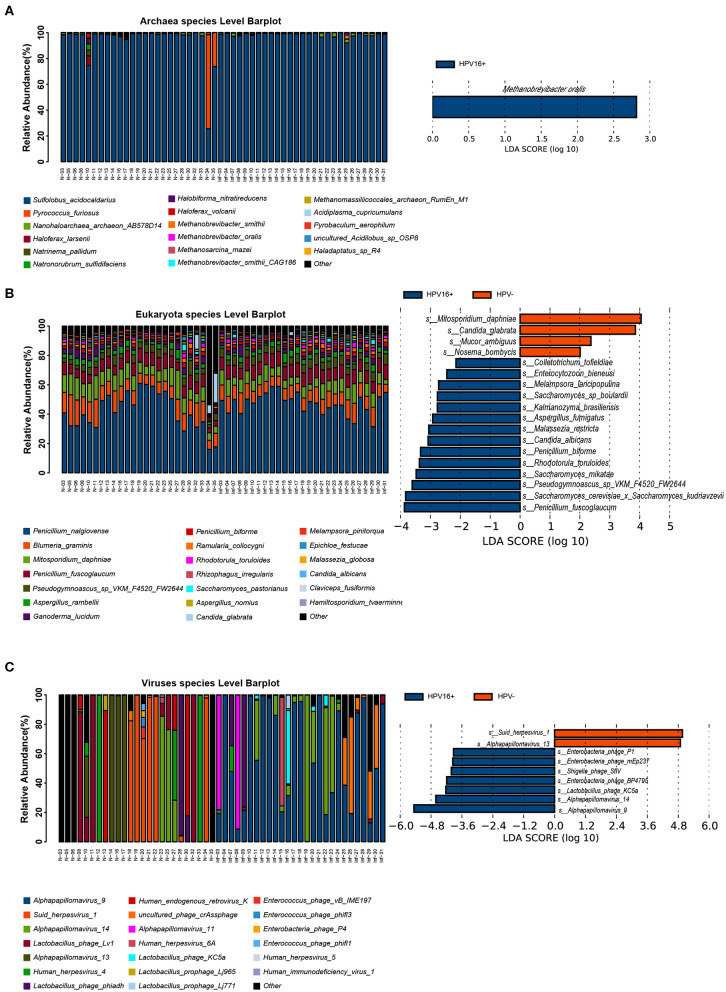
Presence of non-bacterial microbial taxa, archaea (**A**, left), eukaryote (**B**, left), and viruses (**C**, left) within the vaginal microbiome. Histogram of the LDA scores computed for species differentially abundant between HPV16-positive women and healthy controls, archaea (**A**, right), eukaryote (**B**, right), and viruses (**C**, right) biomarkers are shown. The LDA scores (log10) > 2 are listed.

To illustrate the presence of non-bacterial taxa and markers, we performed LEfSe analysis between the two groups in archaea, eukaryote and viruses taxa within the vaginal microbiome (Figure 4) and identified several non-bacterial biomarkers such as *Methanobrevibacter oralis* (archaea), *Candida albicans* (eukaryote), and *Alpha papillomavirus 9* (virus) were enriched in HPV16-positive women, suggesting that non-bacterial taxa can also be associated with HPV16 infection which cannot be ignored.

**Figure 5:**
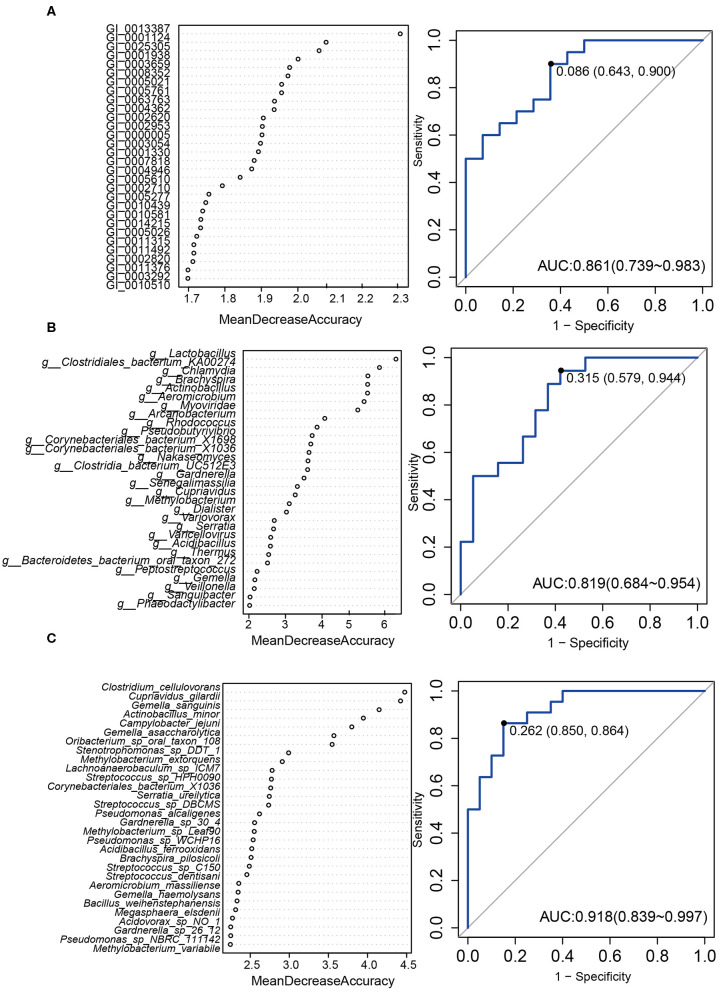
A predictive model of importance based on the gene/genus/species-level abundance profile using random forests (RF). The relative importance of each gene **(A)**/genus **(B)**/species **(C)** in the predictive random forest model using the mean decreasing accuracy. ROC curve generated by the RF using 12 genes **(A)**/17 genus **(B)**/7 species **(C)** in the vaginal microbiota. The plots shown in the ROC represent the corresponding optimal threshold.

Then we constructed a random forest ensemble learning method to distinguish HPV16-positive women from controls using three types of biomarkers: 12 genes (Figure 5A), 17 genera (Figure 5B) and 7 species (Figure 5C). All three of the classifiers based on vaginal microbiome were highly predictive of HPV16-positive status, with the predictive power of 0.861, 0.819, and 0.918, respectively, in ROC analysis, respectively (Figure 5).

### Vaginal Microbial Genes Associated With HPV16 Infection

**Figure 6:**
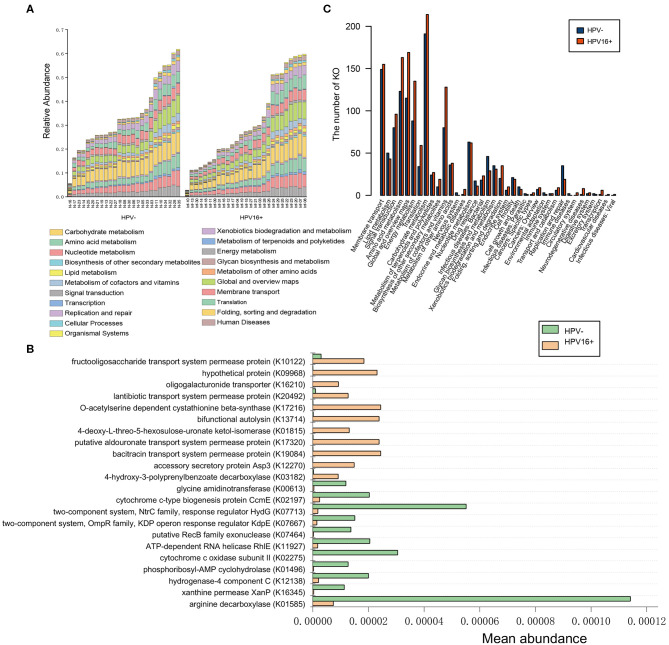
Functional predictions for the vaginal microbiome of the HPV16-positive and control groups. **(A)** The abundance of each sample at level 2 metabolic pathway. **(B)** The KOs with significantly different abundances in the vaginal microbiome identified by Metastats analysis (P, FDR < 0.05). **(C)** Comparison between the HPV16-positive group-enriched and the control-enriched KO markers on level 2 of the KEGG functional category.

A metagenome-wide association study (MGWAS) was performed to identify genes that contributing to the altered gene composition in HPV16-positive women. We annotated the identified genes using the KEGG functional database (V.59) to investigate the certain functional difference between the HPV16-positive and control microbiome. At the level in 1&2 KEGG classification, the HPV16-positive and control metagenome showed a comparable functional configuration. Not surprisingly, carbohydrate metabolism, amino acid metabolism, translation, and membrane transport took up the most number of genes (Figure 6A). We identified 378 KEGG (Kyoto Encyclopedia of Genes and Genomes database) orthologs (KOs) that are involved in 88 pathways were significantly different between the HPV16-positive and controls using Metastats analysis (*p* < 0.05, Supplementary Table 4). KO is a classification system of KEGG proteins or enzymes. The proteins with highly similar sequences and similar functions on the same pathway are grouped. Particularly, a total of 22 KOs were identified with significantly different abundances in the vaginal microbiome between the HPV16-positive and control group (FDR, *P* < 0.05; Figure 6B). Then we annotated the statistically different KOs to the corresponding metabolic pathways, and found that the most prevalent pathways among the HPV16-positive women were those involved in carbonhydrate metabolism, global and overview maps, amino acid metabolism, energy metabolism, membrane transport, and signal transduction. A minority of those were elevated in controls such as glycan biosynthesis and metabolism, and replication and repair (Figure 6C).

### Evaluating HPV16 Infection Biomarkers Using Targeted qPCR

**Figure 7:**
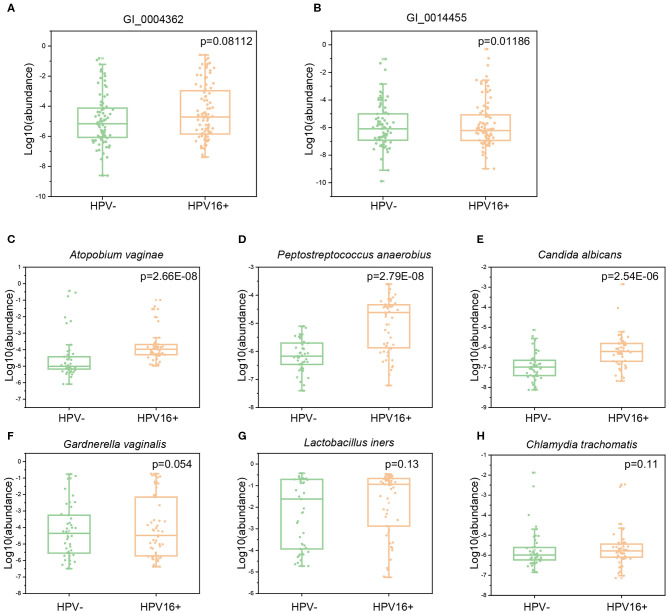
The abundance of gene and species markers in validation cohort by qPCR. Abundance of two gene markers (GI_0004362, C69 family dipeptidase from Gardnerella vaginalis; GI_0014455, GBSi1, group II intron, maturase from multispecies) were measured in validation cohort of 88 cases and 81 controls **(A,B)**. Abundance of six species, Atopobium vaginae **(C)**, Peptostreptococcus anaerobius **(D)**, Candida albicans **(E)**, Gardnerella vaginalis **(F)**, Lactobacillus iners **(G)** and Chlamydia trachomatis **(H)** were measured in a subset of validation cohort (53 cases and 45 controls). The y-axis represents the relative abundance of the corresponding genes and species in all samples. Statistical comparison by Wilcoxon rank-sum test.

To verify whether gene abundances identified by metagenomics sequencing and qPCR are comparable, we randomly selected two HPV16-positive enriched gene markers and measured their abundances in a subset of exploratory cohort (10 controls and 23 cases). Quantification by qPCR and metagenomic sequencing showed strong correlations (Pearson *r* = 0.72, 0.86, respectively) (Supplementary Figure 2), suggesting that both methods are reliable. Then, we measured the abundance of these gene and microbial markers using qPCR in the independent validation cohort (169 vaginal samples; 88 cases and 81 controls). Two gene markers enriched in HPV16-positive women (GI_0004362, C69 family dipeptidase from *Gardnerella vaginalis*; GI_0014455, GBSi1, group II intron, maturase from multispecies) were also enriched in cases of validation cohort (Wilcoxon rank-sum test, *p* = 0.08112 and 0.01186), respectively (Figures 7A,B). We also measured six species enriched in the HPV16-positive group using a subset of validation cohort (98 vaginal samples; 53 cases and 45 controls). Three species were significantly abundant in the HPV16-positive group, including *Atopobium vaginae* (*p* = 2.66E-08, Wilcoxon-rank sum test), *Peptostreptococcus anaerobius* (*p* = 2.79E-08, Wilcoxon-rank sum test) and *Candida albicans* (*P* = 2.54E-06, Wilcoxon-rank sum test) (Figures 7C–E). But, the differences of the other three species enriched in HPV16 positive women did not reach statistical significance, including *Gardnerella vaginalis* (*p* = 0.054, Wilcoxon-rank sum test), *Lactobacillus iners* (*p* = 0.13, Wilcoxon-rank sum test), and *Chlamydia trachomatis* (*p* = 0.11, Wilcoxon-rank sum test) (Supplementary Table 6 and Figures 7F–H).

## Discussion

As an important part of the female lower genital tract local environment, the vaginal microbiome has been paid increasing attention for its potential role in female reproductive health (Chen et al., 2017; Smith and Ravel, 2017; van de Wijgert, 2017; Anahtar et al., 2018). Due to the technical limitation, researches in vaginal microbiota have long been restricted to a very small number of culturable bacteria. Hence, the huge amount of fastidious and uncultivable bacteria, viruses, and fungi in the vagina has been ignored. The application of sequencing technology has completely changed this situation (Quince et al., 2017).

In this study, we utilized shotgun metagenomic sequencing to describe the profiling of vaginal microbiota associated with HPV16 infection. On this basis, taxonomic analysis found that, at the phylum level, Firmicutes was dominant in both groups, but the abundance in the HPV16-positive women was much lower than that in the controls, and other phyla therefore proliferated, suggesting that some bacteria (e.g., Actinobacteria and Fusobacterium) that are originally suppressed by the dominant bacteria were grown in HPV16-positive women. Accordingly, the abundance of *Lactobacillus* (Firmicutes) was lower in HPV16-positive women than that in controls. It has been revealed that *Lactobacillus* adheres to the surface of vaginal epithelial cells, thereby preventing the adhesion of other pathogenic bacteria or viruses; further, *Lactobacillus* produces lactic acid by decomposing glycogen on vaginal epithelial cells, maintains a low pH environment in the vagina, and produces antimicrobial compounds such as bacteriocin, hydrogen peroxide to inhibit the growth of other microorganisms, thereby maintaining normal vaginal microecology (Boris and Barbés, 2000; Tachedjian et al., 2017). Along with the reduction of *Lactobacillus*, a large number of opportunistic pathogens and pathogenic bacteria were increased in HPV16-positive women, including *Gardnerella vaginalis, Mobiluncus curtisii, Peptostreptococcus anaerobius, Fusobacterium nucleatum, Prevotella*, and oral pathogens-*Parvimonas micra*. It has been known that virulence factors such as adhesion, cytotoxin (vaginolysin) (Randis et al., 2013; Nowak et al., 2018), and sialidases (Lewis et al., 2013) produced by *Gardnerella* participate in the dysbiosis. A study found that the sialidase-encoding gene was enriched in HPV-positive patients (Di Paola et al., 2017). *Gardnerella* may utilize sialidase activity and vaginolysin to degrade mucus to assist HPV viruses to enter easily into the host's cells, but further study is needed. *Prevotella* sp. is another bacteria producing sialidase (Briselden et al., 1992). *Mobiluncus curtisii* is usually identified as a BV-related bacteria, a recent study found its association with increased risk of spontaneous preterm delivery (Elovitz et al., 2019). *Peptostreptococcus anaerobius* and *Fusobacterium nucleatum* have been reported to promote tumorigenesis in colorectal cancers (Kostic et al., 2013; Tsoi et al., 2017; Long et al., 2019). In addition, we identified the over-representation of oral pathogen-*Parvimonas micra* in the vaginal fluid from HPV16-positive group, suggesting there may be a route of HPV16 infection by oral-vagina dissemination. Thus, our findings suggest that HPV infection is usually accompanied by mixed infections of various pathogens, and the maintenance of stable vaginal microecology may be a potential pathway to prevent or eliminate HPV infection.

Further, we analyzed the metabolic pathways of the KOs with significant differences between the HPV16-positive women and controls, and found that most of the metabolic pathways were enriched in the HPV16-positive women, such as carbohydrate metabolism, amino acid metabolism, membrane transport, and signal transduction. Active metabolism of the vaginal microbiota may provide a favorable microenvironment for HPV and other pathogen survival. In addition, a minority of microbial metabolic pathways such as glycan biosynthesis and metabolism, replication, and repair were found to be enriched in controls. The best-understood cell-cell interaction in which glycan participate is immunoregulatory activity (Schnaar, 2016). Nowhere is the importance of glycan recognition better understood than in infection and immunity (Raman et al., 2016), and knowledge in this area has already led to glycan mimetic anti-infective and anti-inflammatory drugs (Li et al., 2018). Thus, our results suggest that a stronger glycan biosynthesis and metabolism ability in the normal vaginal microbiome may be one of the mechanisms to resist dysbacteriosis and HPV infection.

We also successfully constructed a random forest model that may be used for distinguishing HPV16 infection and not by generating three types of biomarkers: 12 genes, 17 genera, and 7 species, and found the predictive powers of 0.861, 0.819 and 0.918 in ROC analysis, respectively, suggesting that vaginal microbial targeted biomarkers might be a concomitant signature of HPV infection. However, the identification of the random forest model using a validation cohort is needed.

There were some limitations in the study. Firstly, the diversity in each sample varied drastically due to various factors such as host characteristics (i.e., immune and genetic factors), personal hygiene and sexual behaviors, and hormonal cycling, which might influence the results as confounding factors. Secondly, we couldn't clarify the detailed roles of the vaginal microbiota in the high-risk HPV infection from this cross-sectional study. Longitudinal studies focus on the dynamic fluctuations of vaginal microbiota among high-risk HPV-infected women will provide clues to evaluate which condition precedes the other.

Taken our results together, we found an altered composition of vaginal microbiome in HPV16-positive women, such as decreased *Lactobacillus* and increased *Gardnerella*, including other opportunistic pathogens, with an active metabolism, suggesting that vaginal microbiota dysbiosis that accompanies HPV infection may contribute to HPV persistent infection, even lesion progression.

## Data Availability Statement

Metagenomic sequencing data for all the samples have been deposited in NCBI with the accession number of PRJNA576566.

## Ethics Statement

The studies involving human participants were reviewed and approved by the Ethics Committee of the Women's Hospital, Zhejiang University School of Medicine. The patients/participants provided their written informed consent to participate in this study.

## Author Contributions

WL, QY, YW, XWa, and XX conceived the idea. QY and YW collected and extracted all the samples. QY, YW, XWe, and JZ performed the sequencing and data analysis. QY grafted the manuscript. All authors read, corrected, and approved the final manuscript.

## Conflict of Interest

The authors declare that the research was conducted in the absence of any commercial or financial relationships that could be construed as a potential conflict of interest.
