# Supplementary material for: The Alterations of Vaginal Microbiome in HPV16 Infection as Identified by Shotgun Metagenomic Sequencing
Source: Front Cell Infect Microbiol. 2020 Jun 23;10:286. doi: 10.3389/fcimb.2020.00286 (PMC7324666; doi:10.3389/fcimb.2020.00286)
Supplement: Supplementary file 7 [file Data_Sheet_1.DOCX]

**
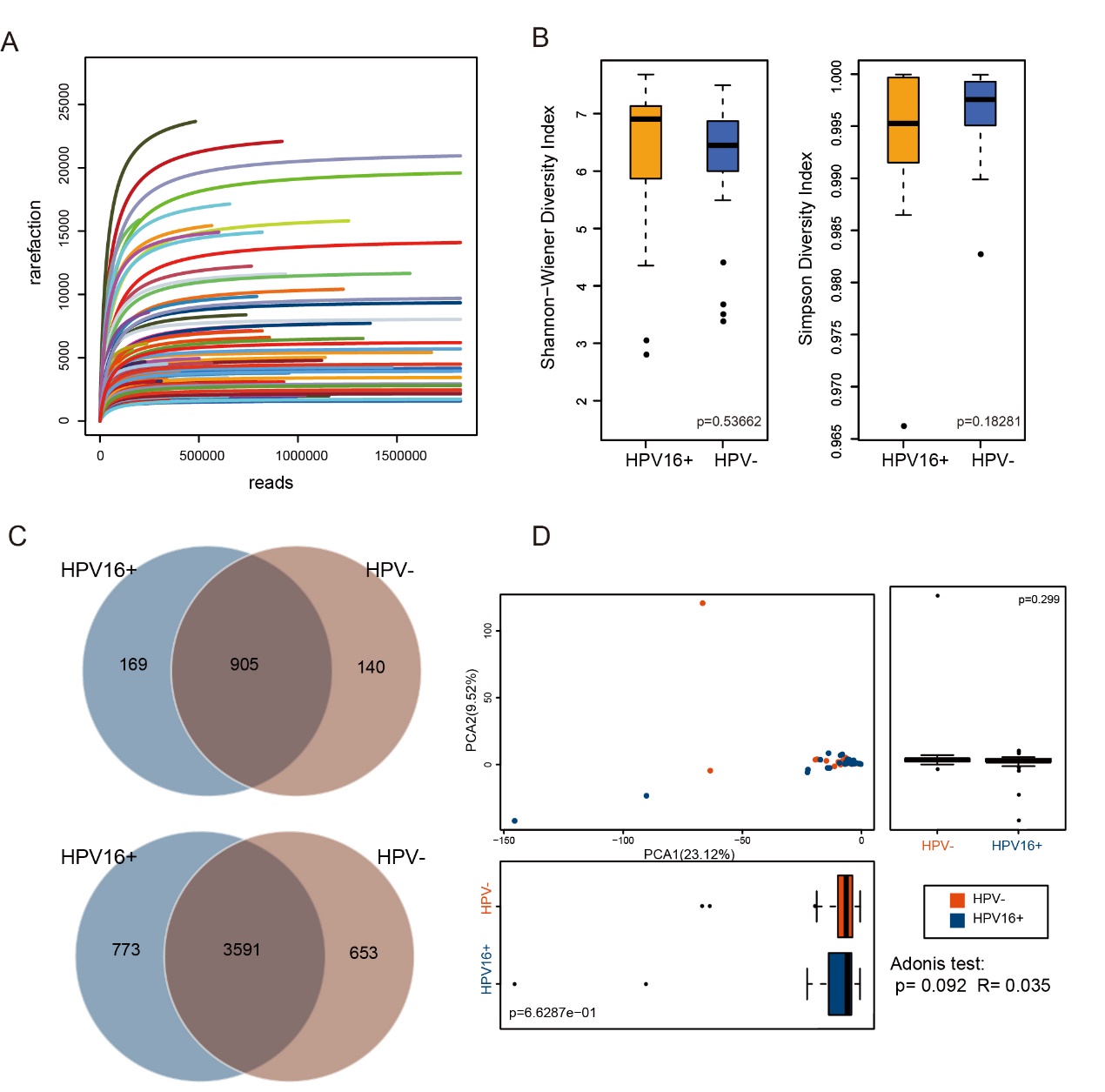
**

**Supplementary Figure 1.** Variations in HPV16 infection associated vaginal microbiome community. (A) Species rarefaction curve showing the sequencing depth of vaginal microbiome in all samples. The horizontal coordinate represents the number of reads, and the ordinate is the number of species. Different colors represents different samples. (B) Shannon-Wiener Index and Simpson Diversity Index between patients (n=27) and control (n=25). (C) Venn diagram show the shared and unique genera (the image above) and species (the image below) of HPV negative controls and HPV16-infected patients. (D) Unsupervised principal component analysis of Bray-Curtis distance showing the stratification of patients from control samples by microbiota compositional profile. Control, n=25; HPV16-infected patients, n=27. PC1 and PC2 represent the top two principal coordinates that reflect most of the diversity, given as a percentage. Groups were compared using Mann-Whitney U test.
