# Supplementary material for: The Alterations of Vaginal Microbiome in HPV16 Infection as Identified by Shotgun Metagenomic Sequencing
Source: Front Cell Infect Microbiol. 2020 Jun 23;10:286. doi: 10.3389/fcimb.2020.00286 (PMC7324666; doi:10.3389/fcimb.2020.00286)
Supplement: Supplementary file 8 [file Data_Sheet_2.DOCX]

**
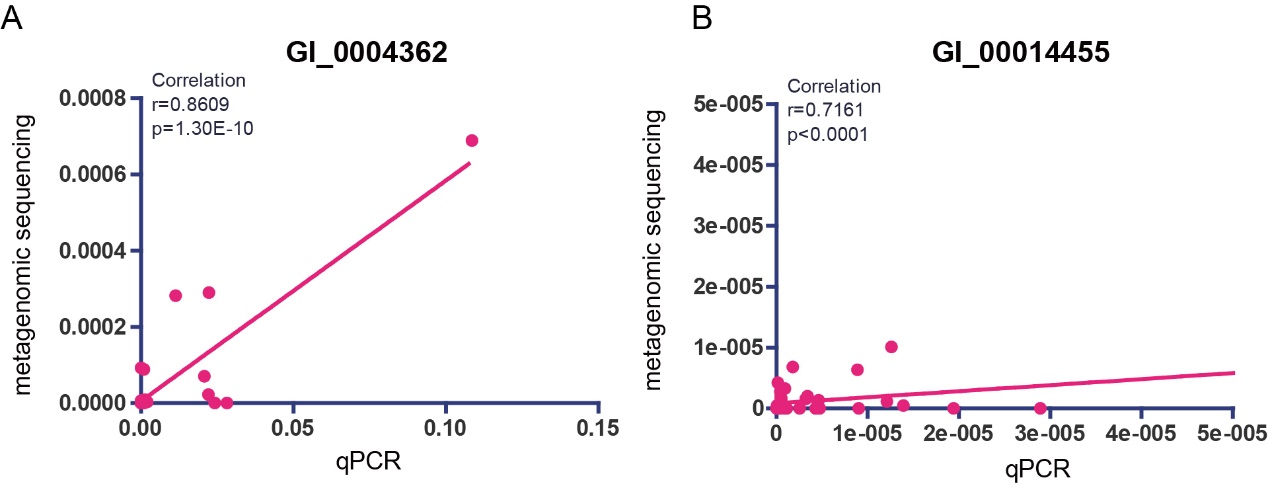
Supplementary Figure S2.** Correlation between quantification by the metagenomic approach verses quantitative polymerase chain reaction (qPCR) for two gene markers (n=33).
